# The distribution of *HLA-A*, *-B*, and *-DRB1* alleles and haplotypes in inhabitants of Guizhou Province of China^☆^

**DOI:** 10.1016/S1674-8301(11)60044-4

**Published:** 2011-09

**Authors:** Qinqin Pan, Su Fan, Xiaoyan Wang, Xing Zhao, Meng Pan, Chengya Wang, Jie Shen

**Affiliations:** HLA Laboratory, the First Affiliated Hospital of Nanjing Medical University, Nanjing, Jiangsu 210029, China.

**Keywords:** human leukocyte antigen, allele, haplotype, linkage, disequilibrium, Guizhou

## Abstract

The present study was aimed to analyze the frequencies of human leukocyte antigen (*HLA*)-*A*, -*B*, and -*DRB1* alleles and *A*-*B*-*DRB1*, *A*-*B*, *A*-*DRB1* and *B*-*DRB1* haplotypes in inhabitants of Guizhou province, China. All samples were typed in the *HLA*-*A*,-*B*, and -*DRB1* loci using the polymerase chain reaction-reverse sequence specific oligonucleotide probe (PCR-rSSOP) method and *HLA* polymorphisms were analyzed. A total of 18 *HLA*-*A*, 31 *HLA*-*B*, and 13 *HLA*-*DRB1* alleles were found in the Guizhou population. The first two frequent alleles in the *HLA*-*A*, -*B*, and -*DRB1* loci were A*11(30.72%) and A*02(30.65%), B*40(16.27%) and B*46(16.27%), and DRB1*09(15.91%) and DRB1*15(13.51%), respectively. The most common haplotype was A*02-B*46-DRB1*09(5.59%) in *A*-*B*-*DRB1*, A*02-B*46(11.73%) in *A*-*B*, B*46-DRB1*09(7.49%) in *B*-*DRB1*, and A*02-DRB1*09(8.08%) in *A*-*DRB1*. Some haplotypes with strong linkage disequilibrium (LD) were found not only in the common haplotypes, such as A*33-B*58, B*30-DRB1*07, and B*33-DRB1*03, but also in the rare haplotypes, such as A*01-B*37, B*37-DRB1*10, and A*01-DRB1*10. Guizhou inhabitants shared some characteristics of the Southern Chinese population but also had their own unique features. Overall, *HLA* polymorphism in Guizhou population was more consistent with that of Chengdu population than that of other populations in China.

## INTRODUCTION

Guizhou province is located in the southwest of China. It adjoins Sichuan province and Chongqing municipality to the north, Yunnan province to the west, Guangxi province to the south and Hunan province to the east. Guizhou is a mountainous province; however, while it is mountainous in the west, the eastern and southern regions are relatively flat. Guizhou covers an area of over 176,000 square kilometers with a total population of more than 35,245,000. Guizhou is one of the provinces that contain the greatest number of minority groups. There are 49 ethnic groups living there, with minorities making up about 38% of the population and their compositions rank third in China after Yunnan Province and Xinjiang Autonomous Region.

Human leukocyte antigen (*HLA*) genes are located at the short arm of chromosome 6 within a region of a few million base pairs. *HLA* is an extremely polymorphic genetic system and its constituent gene products play important roles in the immune response for unrelated hematopoietic stem cell transplantation[1],[2]. *HLA* haplotype analysis is important for identifying appropriate donors, and the most important clinical application of *HLA* haplotype has been the selection of suitable donors in transplantation[3]. *HLA* matching at the haplotype level may have a higher likelihood of matching at other loci than matching merely at the allele level[4]. On the other hand, an accurate and adequate characterization of the distribution of *HLA* alleles and haplotypes at the population level may have been lagging. Hence, determination of the distribution of *HLA* alleles and haplotypes in different populations is necessary for selecting acceptable unrelated donors for patients. With the development of the Chinese Marrow Donor Program (CMDP), more and more *HLA* typing data have become availabe, which provides us a good chance for analyzing *HLA* polymorphism. In addition, *HLA* typing technology has developed rapidly with the development of CMDP, and PCR technology has been applied in the DNA- based *HLA* typing method. Techniques available for DNA typing include sequence specific oligonucleotide probes (SSOP), sequence-specific primers (SSP) and sequence-based typing (SBT). However SBT technology requires expensive equipment, and the first two techniques give rise to flexibility with respect to the desired level of resolution depending on the number of oligonucleotide probes or primers used[5],[6].

In this paper, we examined the frequencies of *HLA*-*A*, -*B*, and -*DRB1* alleles in a total of 2,879 persons residing in the Guizhou province of China. Furthermore, we estimated the frequencies of two or three locus haplotypes and the linkage disequilibrium test between two pairs of loci.

## MATERIALS AND METHODS

### Subjects

Analysis included 2,879 donors recruited into the CMDP Guizhou Branch from August 2006 to December 2007. All donors, regardless of ethnic groups, were included in this study (Han 85%; Miao, Dong, and Buji *etc* 15%, aged from 20-45 years) and were typed for *HLA*-*A*, *HLA*-*B* and *HLA*-*DRB1* in our laboratory. The experiment protocol was approved by the Institutional Review Board of the First Affiliated Hospital of Nanjing Medical University, and all subjects signed informed consent.

### HLA typing

All donors were typed using PCR-reverse SSOP (PCR-rSSOP) method for *HLA*-*A*, -*B* and -*DRB1* using commercial kits (LABtype rSSO Typing Test, lot# A007, B009, DRB0010, OLI, CA, USA). LABType® SSO is a reverse SSO (rSSO) DNA typing method using SSOP and color-coded microspheres to identify *HLA* alleles. First, genomic DNA was isolated from whole blood using the salting-out procedure with commercial kits (DNA Isolation Kit, Dynal Biotech, Brown Deer, Wisconsin, USA). The appropriate DNA concentration was 20-40 ng/µL and the relatively good purity of *A*_260_/*A*_280_ was 1.6-1.8. Then, the sample DNA was subjected to PCR amplification (PE9700, Thermo cycler Life technologies, USA) in a 10 µL reaction volume, with the PCR run at 96°C for 3 min, 96°C for 20 s, 60°C for 20 s, and 72°C for 20 s, for 5 cycles, and 96°C for 10 s, 60°C for 15 s, and 72°C for 20 s for 30 cycles followed by 72°C for 10 min and stored at 4°C forever. After amplification, the PCR products were denatured and neutralized with acids and bases, and then the PCR products were hybridized with the corresponding locus beads at 60°C for 15 min, which were washed three times using the washing buffer. Then, streptavidin conjugated phycoerythrin (SAPE) was reacted with the products for 5 min at 60°C, and following washing, the products were detected using the Luminex 200 after being suspended with 60 µL washing buffer. Fluorescence signals were identified by the laser Luminex 200 (Luminex, USA), and lastly the HLA typing was obtained from the software HLAtools.

### Statistical analysis

HLA allele frequencies (AF) were determined for each allele in donors using the formula: AF (%) = (n/2N)×100%, where *n* indicates the sum of a particular allele and *N* indicates the total number of individuals.

The maximum-likelihood haplotype frequencies, the Hardy-Weinberg equilibrium, and the linkage disequilibrium (LD) test were computed by the software Arlequin 3.01 using the expectation-maximization (EM) algorithm. Hardy-Weinberg exact tests were performed on all samples for each of the three HLA loci. The EM algorithm is a very general principle for handling missing data in statistical analysis. This algorithm has been described in detail somewhere as applied to estimation of multilocus haplotype frequencies. EM is an iterative method which alternates between performing an expectation (E) step, which computes the expectation of the log-likelihood evaluated using the current estimate for the latent variables, and a maximization (M) step, which computes parameters maximizing the expected log-likelihood found on the E step. These parameter-estimates are then used to determine the distribution of the latent variables in the next E step[7],[8].

The parameters reflecting LD intensity of D, D′, and r^2^, and chi-square value given by Arlequin were also shown and the mathematic definitions of D, D′ and r^2^ were given in detail elsewhere[9].

## RESULTS

### Hardy-Weinberg equilibrium examination

Hardy-Weinberg exact tests were performed on the three HLA loci. The observed, expected homozygosities and the statistical *P* value are given in ***Table 1***. The results showed that the *P* values at the three loci were all more than 0.05. The *P* value was used to measure the magnitude of the deviation in a population sample, if a *P* value greater than 0.05, indicated that the population were consistent with Hardy-Weinberg equilibrium[10], which meant that the population was random and the sample size was adequately large[11],[12].

**Table 1:** The Hardy-Weinberg equilibrium of *HLA*-*A*, -*B*, and -*DRB1* loci in Guizhou population

Locus	Genot	bs.Heter.	Exp.Heter.	*P*	SD
*A*	2,879	0.774	0.774	0.131	0.001
*B*	2,879	0.911	0.903	0.263	0.000
*DRB1*	2,879	0.898	0.900	0.699	0.001

### Allele frequencies

A total of 18 *HLA*-*A*, 31 *HLA*-*B*, and 13 *HLA*-*DRB1* alleles were found in Guizhou population. In the *HLA*-*A* locus, A*11 was the most frequent allele in the present study with a frequency of 30.72%, followed by A*02(30.65%), A*24(17.07%), and A*33(7.43%). In the *HLA*-*B* locus, B*40 and B*46 were ranked as the first two frequent alleles with the same frequency of 16.27%, followed by B*15 (13.89%), B*13(9.66%), B*51(6.34%) and B*58(6.32%). In the *HLA*-*DRB1* locus, DRB1*09 was the most common one (15.91%), followed by DRB1*15(13.51%), DRB1*12 (13.06%), DRB1*04 (10.44%) and DRB1*14 (9.34%). In addition, some *HLA* alleles were found to be very rare in the Guizhou population. For example, A*25(0.02%) and A*36(0.02%) in the *HLA*-*A* locus, and B*53(0.02%) and B*59(0.02%) in the *HLA*-*B* locus. Besides, some *HLA* alleles were not detected at all, such as A*43, B*82 and B*83. The frequencies of *HLA*-*A*, -*B*, and -*DRB1* alleles are described in ***Table 2***. The *HLA* allele distribution (***Table 3***) showed that the majority of Guizhou population harbored the most common alleles. There were three alleles in the *HLA*-*A* locus (over 10%) with a cumulative frequency of 78.44%, three alleles in the *HLA*-*B* locus with a cumulative frequency of 46.43%, and four alleles in the *HLA*-*DRB1* locus with a cumulative frequency of 52.92%. Overall, the alleles with frequencies more than 1% in the *HLA*- *A*, -*B*, and -*DRB1* loci made up 90% of the total population.

### Haplotype frequencies and linkage disequilibrium (LD)

**Table 2:** Frequencies of *HLA*-*A*, -*B*, and *DRB1* alleles in Guizhou population

Allele group	Allele frequency (%)	Allele group	Allele frequency (%)	Allele group	Allele frequency(%)
HLA-A*		B*14	0.07	B*58	6.32
A*01	1.98	B*15	13.89	B*59	0.02
A*02	30.65	B*18	0.59	B*67	0.50
A*03	1.72	B*27	1.77	B*81	0.03
A*11	30.72	B*35	4.18	Blank	0.06
A*23	0.19	B*37	0.88	HLA-DRB1*	
A*24	17.07	B*38	3.30	DRB1*01	2.17
A*25	0.02	B*39	2.10	DRB1*03	5.03
A*26	2.29	B*40	16.27	DRB1*04	10.44
A*29	0.75	B*41	0.05	DRB1*07	5.14
A*30	3.26	B*44	1.80	DRB1*08	7.29
A*31	2.67	B*45	0.03	DRB1*09	15.91
A*32	0.55	B*46	16.27	DRB1*10	1.59
A*33	7.43	B*47	0.03	DRB1*11	5.95
A*34	0.06	B*48	1.78	DRB1*12	13.06
A*36	0.02	B*49	0.10	DRB1*13	5.69
A*68	0.46	B*50	0.35	DRB1*14	9.34
A*69	0.07	B*51	6.34	DRB1*15	13.51
A*74	0.05	B*52	2.01	DRB1*16	4.84
Blank	0.04	B*53	0.02	Blank	0.04
HLA-B*		B*54	2.57		
B*07	2.47	B*55	3.96		
B*08	0.90	B*56	0.90		
B*13	9.66	B*57	0.78		

(N = 2,879×2)

The haplotypes of *A*-*B*-*DRB1*, *A*-*B*, *B*-*DRB1*, and *A*-*DRB1* occurring at frequency over 1% are summarized in ***Table 4***. The most common *A*-*B*-*DRB1* haplotype in this study was A*02-B*46-DRB1*09 with a frequency of 5.59%, followed by A*33-B*58-DRB1*03 (2.80%), A*30-B*13-DRB1*07 (2.23%) and A*02-B*46-DRB1*14 (2.10%). The most common *A*-*B* haplotype was A*02-B*46 with a frequency of 11.73%, followed by A*11-B*15 (6.89%), A*11-B*40 (6.19%), A*33-B*58 (5.10%), and A*24-B*40 (5.01%). In the *B*-*DRB1* haplotype, B*46-DRB1*09 was the most common with a frequency of 7.49%, followed by B*15-DRB1*12 (3.82%), B*58-DRB1*03 (3.47%) and B*46-DRB1*14 (2.94%). In the *A*-*DRB1* haplotype, some common haplotypes could be ranked as A*02-DRB1*09 with a frequency of 8.08%, followed by A*11-DRB1*12 (6.07%), A*11-DRB1*15 (5.68%) and A*11-DRB1*04 (3.57%).

**Table 3:** Distribution of *HLA*-*A*, -*B*, and -*DRB1* genes

Frequency range (%)	*HLA-A*		*HLA-B*		*HLA-DRB1*	
	*n*	Cumulative frequency	*n*	Cumulative frequency	*n*	Cumulative frequency
Total number	18	99.96%	31	99.94%	13	99.96%
> 10%	3	78.44%	3	46.43%	4	52.92%
1%-10%	6	19.35%	13	48.26%	9	47.04%
0.1%-1%	4	1.95%	8	5.00%	0	0
< 0.1%	5	0.22%	7	0.25%	0	0

### Linkage disequilibrium

The results of linkage disequilibrium (LD) test between two pairs loci are summarized in ***Table 5***-***7*** ranked by the LD parameter, r^2^ value. Some strong LD haplotypes were detected between two loci, including the common haplotypes and the rare haplotypes. For example, in the *A*-*B* haplotype, the haplotype with the strongest LD were A*33-B*58 with a frequency of 5.1%, while the haplotype with the second strongest LD was a rare haplotype (A*01-B*37) only with a frequency of 0.68%. In the *A*-*DRB1* haplotype, the first two strongest LD haplotypes were common haplotypes including A*30-DRB1*07 (2.30%) and A*33-DRB1*03 (2.85%). However, the third strongest one was a rare haplotype A*29-DRB1*10 with a frequency of 0.38%. While in the *B*-*DRB1* haplotype, B*37-DRB1*10 with a frequency of 0.76% was ranked as the first strongest LD haplotype, followed by three common haplotypes, which were B*58-DRB1*03 (3.46%), B*46-DRB1*09 (7.49%) and B*13-DRB1*07 (2.85%).

**Table 4:** Common haplotypes in Guizhou population occurring at a frequency over 1%

*A-B-DRB1*	ML-HF (%)	*A-B*	ML-HF (%)	*B-DRB1*	ML-HF (%)	*A-DRB1*	ML-HF (%)
02XX 46XX 09XX	5.59	02XX 46XX	11.73	46XX 09XX	7.49	02XX 09XX	8.08
33XX 58XX 03XX	2.80	11XX 15XX	6.89	15XX 12XX	3.82	11XX 12XX	6.07
30XX 13XX07XX	2.23	11XX40XX	6.19	58XX 03XX	3.47	11XX 15XX	5.68
02XX 46XX 14XX	2.10	33XX 58XX	5.10	46XX 14XX	2.94	11XX 04XX	3.57
11XX 15XX 15XX	1.58	24XX 40XX	5.01	15XX 15XX	2.93	02XX 12XX	3.57
02XX 46XX 08XX	1.47	11XX 13XX	3.92	15XX 04XX	2.88	02XX 15XX	3.53
33XX 58XX 13XX	1.40	02XX 40XX	3.48	13XX 07XX	2.85	02XX 04XX	3.08
11XX 15XX 04XX	1.35	02XX 15XX	3.18	13XX 15XX	2.76	33XX 03XX	2.85
11XX 46XX 09XX	1.27	11XX46XX	2.80	40XX 12XX	2.51	02XX 08XX	2.76
11XX 40XX 12XX	1.22	30XX 13XX	2.78	40XX 15XX	2.45	02XX 14XX	2.53
11XX 40XX 08XX	1.16	24XX 15XX	2.73	40XX 09XX	2.30	24XX 09XX	2.51
24XX 40XX 15XX	1.00	02XX 38XX	2.04	40XX 08XX	2.16	11XX 08XX	2.51
		11XX51XX	1.98	46XX 08XX	2.09	24XX 15XX	2.48
		02XX 13XX	1.94	40XX 11XX	1.93	24XX 12XX	2.47
		02XX 51XX	1.67	58XX 13XX	1.77	11XX 09XX	2.46
		11XX55XX	1.52	40XX 04XX	1.67	11XX 14XX	2.36
		02XX 55XX	1.40	15XX 09XX	1.56	30XX 07XX	2.30
		24XX 51XX	1.39	13XX 12XX	1.54	24XX 14XX	2.25
		24XX 35XX	1.17	51XX 09XX	1.41	24XX 04XX	2.19
		24XX 46XX	1.13	51XX 14XX	1.27	02XX 16XX	2.03
		11XX39XX	1.10	40XX 16XX	1.17	11XX 11XX	1.80
		24XX 54XX	1.07	40XX 14XX	1.16	02XX 11XX	1.73
				46XX 12XX	1.07	11XX 13XX	1.66
						33XX 13XX	1.66
						11XX 16XX	1.53
						24XX 11XX	1.24
						24XX 08XX	1.20

ML-HF: Maximum-likehood haplotype frequencies.

**Table 5:** The relative strongest linkage equilibrium between *HLA*-*A* and -*B*

Haplotype	Observed frequencies (%)	Expected frequencies (%)	D	D′	r^2^	chi-square
A*33-B*58	5.10	0.47	0.046	0.794	0.530	3043.778
A*01-B*37	0.68	0.02	0.007	0.760	0.256	1468.645
A*29-B*07	0.64	0.02	0.006	0.857	0.217	1246.909
A*30-B*13	2.78	0.32	0.024	0.823	0.214	1230.311
A*02-B*46	11.73	4.99	0.075	0.660	0.192	1101.331
A*01-B*57	0.51	0.02	0.005	0.637	0.158	910.244
A*23-B*49	0.05	0	0.001	0.499	0.136	780.135
A*23-B*45	0.17	0	0	0.499	0.045	259.864
A*24-B*40	5.01	2.77	0.027	0.198	0.037	213.080
A*23-B*41	0.02	0	0	0.332	0.030	172.608
A*11-B*15	6.89	4.26	0.027	0.280	0.029	164.227
A*32-B*44	0.16	0.55	0.002	0.300	0.027	156.994
A*02-B*58	0.23	1.93	0.017	-0.892	0.024	135.708
A*11-B*46	2.80	5.00	-0.026	-0.513	0.023	130.330
A*36-B*57	0.02	0	0	1.000	0.022	0.008
A*33-B*44	0.66	0.13	0.005	0.304	0.021	122.448
A*02-B*38	2.04	1.01	0.011	0.504	0.020	112.472
A*03-B*44	0.29	0.03	0.002	0.146	0.020	116.798

**Table 6:** The relative strongest linkage equilibrium between *HLA*-*A* and -*DRB1*

Haplotype	Observed frequencies (%)	Expected frequencies (%)	D	D′	r^2^	chi-square
A*30-DRB1*07	2.30	0.17	0	0.697	0.303	1739.976
A*33-DRB1*03	2.85	0.37	0.025	0.537	0.190	1094.590
A*29-DRB1*10	0.38	0.01	0.040	0.504	0.118	675.721
A*01-DRB1*10	0.58	0.03	0.006	0.357	0.102	588.272
A*02-DRB1*09	8.08	4.87	0.038	0.349	0.052	298.950
A*33-DRB1*13	1.67	0.42	0.013	0.537	0.049	279.805
A*11-DRB1*12	6.07	4.00	0.025	0.276	0.026	147.557
A*02-DRB1*03	0.36	1.54	0.012	-0.808	0.015	87.747
A*11-DRB1*15	5.68	4.16	0.018	0.191	0.013	74.271
A*11-DRB1*07	0.38	1.58	0.012	-0.736	0.013	74.900
A*02-DRB1*13	0.56	1.74	-0.010	-0.600	0.010	54.862

**Table 7:** The relative strongest linkage equilibrium between *HLA*-*B* and -*DRB1*

Haplotype	Observed frequencies (%)	Expected frequencies (%)	D	D′	r^2^	chi-square
B*37-DRB1*10	0.76	0.01	0.010	0.860	0.410	2342.480
B*58-DRB1*03	3.46	0.32	0.030	0.670	0.360	2040.560
B*46-DRB1*09	7.49	2.59	0.050	0.400	0.150	877.110
B*13-DRB1*07	2.85	0.50	0.020	0.520	0.140	791.810
B*07-DRB1*01	0.84	0.05	0.010	0.390	0.130	766.340
B*08-DRB1*03	0.76	0.05	0.010	0.840	0.120	696.090
B*57-DRB1*07	0.53	0.04	0	0.670	0.070	377.250
B*58-DRB1*13	1.77	0.36	0.010	0.260	0.060	356.830
B*15-DRB1*12	3.82	1.81	0.020	0.200	0.040	207.150
B*07-DRB1*10	0.45	0.04	0	0.250	0.040	234.870
B*15-DRB1*04	2.88	1.45	0.020	0.190	0.030	154.070
B*13-DRB1*15	2.76	1.31	0.020	0.190	0.030	146.350

### Comparison of the frequent alleles between Guizhou province and other populations

The first three frequent alleles in the *HLA*-*A*,-*B*, and -*DRB1* loci in Guizhou province and other populations were obtained from the previous studies: the provinces of Shanxi[13], Henan[14], Jiangsu[15], Hunan[16], and Hainan[17]. As it can be seen from ***Table 8***, A*11, A*02, and A*24 were more frequent in Guizhou than in other provinces in China, regardless of whether the population was the northern Chinese or southern Chinese. A*11 was more frequent in southern Chinese than the northern Chinese and A*02 was less frequent in Hunan and Hainan provinces than in the northern Chinese. In the the *HLA*-*B* locus, B*40 and B*46 were more frequent in Guizhou than in other provinces in China. In the *HLA*-*DRB1* locus, DRB1*12 in Guizhou was the most frequent allele among the six provinces, and the frequency of DRB1*15 was between that of the northern Chinese and southern Chinese.

**Table 8:** The first three frequent alleles in *HLA*-*A*,-*B*, and -*DRB1* loci in Guizhou province and other populations in China

Allel	Guizhou	Shanxi	Henan	Jiangsu	Hunan	Hainan
A*11	30.72	17.93	16.66	18.56	20.88	18.35
A*02	30.65	29.26	29.09	29.55	19.64	15.45
A*24	17.07	16.12	15.70	16.79	12.01	11.03
B*40	16.27	12.98	13.15	13.58	15.31	10.78
B*46	16.27	7.15	6.24	9.41	11.86	7.55
B*15	13.89	13.84	13.70	14.40	9.54	8.55
DRB1*09	15.91	12.85	13.02	16.15	13.01	7.56
DRB1*15	13.51	16.77	17.86	13.09	9.55	9.50
DRB1*12	13.06	10.67	9.95	12.22	8.89	8.25

(%)

## DISCUSSION

Guizhou province is in the southwest of China. One would expect that Guizhou presents some of the characteristics of the Southern Chinese population. The common alleles in the Chinese population, such as A*02, A*11, A*24, A*33, B* 40, B*58, B*15, B*46, DRB1*09, DRB1*15, DRB1*12, and DRB1*04, are also found frequently in Guizhou population. Meanwhile, it was shown that inhabitants of Guizhou province exhibit some differences from those of the other provinces of China in some alleles, especially differences from some minority groups, such as Hui, Wa, and Drung[18]-[20]. For example, in locus A, A*02 (30.65%) and A*11 (30.72%) were the first two most frequent alleles in Guizhou province, and there were nearly no differences in the frequencies of two alleles. In Chengdu population[21], A*11 was the most frequent allele with a frequency of 31.50%, and A*02 was the second most frequent allele with a frequency of 31.03%, which is nearly the same as that reported for Guizhou province. The frequency of A*02 in Guizhou province is consistent with that of Southern Chinese, such as that in Jiangsu (A*02, 29.55%)[15] and Shanghai (A*02, 31.34%)[22]. The first four most frequent alleles in Guizhou were in the order of A*11, A*02, A*24 and A*33, and this order is the same as that of Chengdu[21]. In contrast, the first four most frequent alleles in the A locus in Jiangsu and Shanghai are all in the order of A*02, A*11, A*24 and A*33[15], [22]. The first four most frequent alleles in Yunnan province for Han Chinese are in the order of A*24, A*02, A*11 and A*33, which differ more from those in Guizhou population than Jiangsu and Shanghai, although Yunnan province is adjacent to Guizhou province. In the B locus, B*46 (16.27%) is the most common allele in Guizhou population, which is also the most common one in Chengdu(16.3%)[21] and Yunnan (17.9%)[23]. While in Jiangsu[15] and Shanxi[13], B*15 is the most common one. The first four most frequent alleles in the *B* locus in Guizhou are in the order of B*40, B*46, B*15 and B*13, for Han Chinese in Yunnan are B*46, B*15, B*40 and B*13[23], in Jiangsu are B*15, B*40, B*13 and B*46[15]. The four alleles are all the same but in different order. In the *DRB1* locus, DRB1*09 (15.91%) is the most predominant allele, which is also the most predominant one in Jiangsu[15], Shanghai[22] and Chengdu[21] and the first four most frequent alleles in DRB1 locus in Guizhou are in the same order as that in Chengdu, with the same order of DRB1*09, DRB1*12, DRB1*15, and DRB1*04. By comparison of the frequencies of the *HLA* alleles in several provinces, Guizhou is more consistent with Chengdu than other provinces.

*HLA* haplotypes of two and/or three loci have been reported in various worldwide populations[24], which show great difference from the population of Guizhou in haplotype distribution. The most common haplotype in Guizhou is A*02-B*46-DRB1*09, which is the same as that in Chengdu[21]. While in Jiangsu, Shanxi province and Shanghai[15],[13],[22], the most common haplotype is A*30-B*13-DRB1*07. While in Yunnan province, A*24-B*15-DRB1*15 is the most common haplotype with a frequency of 4.1%, followed by A*24-B*46-DRB1*08 (3.5%) and A*24-B*15-DRB1*12(2.9%)[23]. However, these three haplotypes are relatively rare in Guizhou province.

The distribution of the *HLA*-*A*, -*B*, and -*DRB1* alleles (***Table 2***) showed that the majority of Guizhou population harbor the common alleles, which means that most patients for homologous stem cell transplantation (HSCT) would readily find *HLA*-*A*, -*B*, and -*DRB1* matched donors in CMDP Guizhou registry if they carry those common alleles.

*HLA* haplotype estimate is a valuable tool in the management of donor registries. It has been used to project how many donors would be needed to achieve a certain probability of finding an *HLA*-matched donor. Another useful application of the haplotype frequency is to predict the probability that a donor typed at low or intermediate-resolution would match a specific patient at high resolution. Besides, *HLA* haplotype provides valuable information in tracing the source of historical genetic inputs.

In summary, the present study reported *HLA*-*A*, -*B*, and -*DRB1* allele frequencies and haplotype frequencies in Guizhou population. The results would be useful as baseline data for donor selection of hematopoietic stem cell or solid organ transplantation, anthropology studies and *HLA* disease association analysis.
